# Characterization of microsatellite markers for the endangered *Daphne rodriguezii* (Thymelaeaceae) and related species

**DOI:** 10.1002/aps3.11274

**Published:** 2019-07-11

**Authors:** Carlos García‐Verdugo, Juan Carlos Illera, Anna Traveset

**Affiliations:** ^1^ Dept. de Biología Universitat de les Illes Balears Ctra. Valldemossa Km 7.5 Palma 07122 Balearic Islands Spain; ^2^ Jardín Botánico Atlántico – Universidad de Oviedo Avda. del Jardín Botánico 2230 33203 Gijón/Xixón Spain; ^3^ Research Unit of Biodiversity (UO‐CSIC‐PA) Calle Gonzalo Gutiérrez Quirós s/n 33600 Mieres Spain; ^4^ IMEDEA (CSIC‐UIB) Mediterranean Institute for Advanced Studies Calle Miquel Marquès 21 Esporles 07190 Balearic Islands Spain

**Keywords:** *Daphne rodriguezii*, fine‐scale genetic structure, island genetic diversity, paternity analysis, Thymelaeaceae

## Abstract

**Premise:**

The endangered shrub *Daphne rodriguezii* (Thymelaeaceae) is endemic to the Balearic island of Menorca, where fragmentation and severe population decline are ongoing threats to this taxon. We developed a set of microsatellite markers to analyze the fine‐scale genetics of its few extant populations.

**Methods and Results:**

Fifteen microsatellite markers were obtained through Illumina high‐throughput sequencing and tested in two populations. Twelve of these loci showed no evidence of null alleles and were highly polymorphic, with a mean number of 8.3 alleles per locus. Levels of observed and expected heterozygosity ranged from 0.100 to 0.952 and from 0.095 to 0.854, respectively. Seven to nine of these loci were successfully amplified in five other *Daphne* species.

**Conclusions:**

This set of markers provides a useful tool for investigating the factors driving fine‐scale population structure in this threatened species, and it represents a novel genetic resource for other European *Daphne* species.

The genus *Daphne* L. (Thymelaeaceae) comprises ca. 95 species with Eurasian and North African distributions (Brickell and Mathew, 1998). Several studies have highlighted their ecological and medicinal importance (Xu et al., 2011), but the genetic information available for the genus is still scarce, particularly for the 17 European species recognized thus far (e.g., Alonso and Herrera, 2011).

*Daphne* *rodriguezii* Texidor is a perennial shrub endemic to the Balearic island of Menorca. Because the species shows a highly fragmented distribution with severe population decline, the International Union for Conservation of Nature (IUCN) has recently reassessed its conservation status from “Vulnerable” to “Endangered” (Fraga i Arquimbau, 2017). The area of occupancy of *D. rodriguezii* is represented by as few as five main populations (<36 km^2^), all of them restricted to the eastern area of Menorca and the off‐shore islet of Colom (Calviño‐Cancela et al., 2012; Fraga i Arquimbau, 2017). Apart from its interest for biodiversity conservation, the species has been used as a model system to understand key ecological processes such as the impact of loss of dispersal agents (i.e., lizards) on the maintenance of island populations (Traveset and Riera, 2005; Rodríguez‐Pérez and Traveset, 2010). A previous phylogeographic study with plastid and amplified fragment length polymorphism data revealed a strong genetic structure among extant populations, but the unavailability of codominant markers for *D. rodriguezii* limited genetic inferences at the population level (Calviño‐Cancela et al., 2012). Generation of fine‐scale information with microsatellite markers for this species will provide insights on general patterns of heterozygosity across island systems (García‐Verdugo et al., 2015) and will help us understand how dispersal limitation impacts the genetic structure of small island populations (Saro et al., 2019).

## METHODS AND RESULTS

Microsatellite development was conducted by Ecogenics GmbH (Balgach, Switzerland) from genomic DNA extracted from silica‐dried *D. rodriguezii* leaf tissue. An Illumina TruSeq nano DNA library (Illumina, San Diego, California, USA) was prepared following the manufacturer's recommendations and analyzed on an Illumina MiSeq sequencing platform using the Nano 2 × 250 v2 format. A total of 1,507,789 raw reads were processed. The paired‐end reads that passed the Illumina's chastity filter were subject to de‐multiplexing and trimming of Illumina adapter residuals, and subsequently checked with the FastQC v0.11.7 software (Andrews, 2010). Following quality check, paired‐end reads were analyzed with the software USEARCH v10.0.240 (Edgar, 2010), which resulted in 99,945 merged reads that were further screened with the software Tandem Repeats Finder v4.09 (Benson, 1999). Microsatellite sequences with a tetra‐ or trinucleotide of a minimum of six repeat units (or a dinucleotide of at least 10 repeat units) were detected in 4584 merged reads. Primers were designed for 2950 microsatellite regions using Primer3 (Untergasser et al., 2012). From these candidate loci, and with the aim of optimizing marker characterization, we performed an initial step of simple sequence repeat (SSR) polymorphism testing by amplifying 36 loci in a subset of seven *D. rodriguezii* individuals. These loci were screened based on the motif (i.e., trinucleotide SSRs were preferred over dinucleotide SSRs), the size of the amplified fragment (for optimization of the subsequent design of multiplexed reactions), and their successful amplification in all of the samples. Considering the allele size ranges and the apparent lack of null alleles across the seven individuals tested, we finally used 15 out of the 36 loci to evaluate their usefulness in revealing polymorphism with larger (i.e., population level) sample sizes.

In examining the levels of variability revealed by each SSR locus, we were constrained by the conservation status (EN) of the study species. However, we were able to obtain permissions to sample leaf material from two populations representing size extremes (Calviño‐Cancela et al., 2012): (1) the only population with more than 300 mature individuals (population A) and (2) a population with <50 individuals (population B) (Appendix 1). Genomic DNA was extracted using the NucleoSpin Plant II kit (Macherey‐Nagel, Düren, Germany) following the cetyltrimethylammonium bromide (CTAB)–lysis method. PCRs for SSR amplification were set up in 10‐μL reactions, including 1.5 μL of DNA (2–10 ng/μL), 5 μL of 2× Multiplex PCR Master Mix (QIAGEN, Hilden, Germany), and 0.3 μL (0.3 μM) of each primer, with the forward primer labeled with a fluorescent dye (Table 1). Reactions were performed on a G‐Storm GS2 thermal cycler (Somerton Biotechnology Centre, Somerset, United Kingdom) under the following conditions: initial denaturation at 95°C for 15 min; followed by 35 cycles of denaturation at 95°C for 30 s, annealing at 56°C for 45 s, and extension at 72°C for 45 s; and a final extension at 60°C for 30 min.

**Table 1:** Characteristics of 15 microsatellite loci developed in *Daphne rodriguezii*.

Locus^a^	Primer sequences (5′–3′)	Repeat motif	Allele size range (bp)	*A*	Multiplex	Fluorescent dye	GenBank accession no.
Dro012	F: CATAATGCTGACGTGGACGG	(CTT)_9_	237–270	11	1	FAM	MK507747
	R: ATGGAGGCGGGAAACTTAGG						
Dro019	F: CGGAGGGATTCAAACTTGGG	(ATT)_9_	246–258	5	4	FAM	MK507756
	R: TGTTGACTTCAATTTTTGTGCGG						
Dro025	F: TAACGGCATTGCAGGTTTTC	(TA)_26_	157–159	2	5	FAM	MK507759
	R: GGGTGTATAGCCCCTACGTC						
Dro028	F: TAAAAAGCGCCGGACTCAAG	(TCT)_12_	175–190	6	2	FAM	MK507752
	R: AGCTGGTTCCGTACGATGAG						
Dro034	F: TGGCAGTAGACAACATTAGTGG	(TTA)_17_	199–238	12	3	HEX	MK507754
	R: AGACGTGCTGAAGACAGTTC						
Dro035	F: AACATCGATTTCTGTCGCGG	(TA)_16_	204–210	2	4	HEX	MK507760
	R: ACGGGGCTTTTTGTGCATTC						
Dro041	F: GAATCCCAACTGCATCGTGG	(GAT)_14_GG(TGA)_9_	240–261	8	2	FAM	MK507750
	R: TGGGCTTGTCATGGTAAACG						
Dro042	F: AGGGTATTTCGTGGGCTGG	(TTA)_21_	243–318	17	3	FAM	MK507753
	R: ACAATGTAAAAAGCAAGAAATCCAC						
Dro046	F: CCCGCTTTACTTCAGTGTCG	(CAT)_12_	180–204	7	3	FAM	MK507755
	R: ATCGCTAAGATTCGGGTTGC						
Dro048	F: CTCCAAACCATTTCCTGAGTCG	(TA)_14_	218–236	9	2	HEX	MK507751
	R: ACACCACGCCATTTAATTCTCC						
Dro073	F: GACTGACGATGTCTACATGAGC	(AT)_21_	205–217	2	5	HEX	MK507761
	R: GGTGGAGTACAACCATCCTTTG						
Dro078	F: TTATGGGCTTAGAGCCACCG	(AT)_22_	185–223	15	4	FAM	MK507758
	R: AAAGTCGCCACCGGAAAATG						
Dro113	F: TTTGGCTTTGAACCATCCCG	(TCT)_11_	114–132	6	1	FAM	MK507749
	R: GTCCAAACACCAACTATAATGAAAGC						
Dro114	F: ACGCTTCCGCAATATGATCG	(TCT)_10_	198–234	11	1	HEX	MK507748
	R: CAGACGATACTGAGGGGTGG						
Dro124	F: AGAGCTTTCCAAGAATTGATGTAG	(AAT)_20_	233–287	12	5	FAM	MK507757
	R: TACCCATGCACGGAGTGTG						

Note

*A* = number of alleles found across all *D. rodriguezii* samples.

a Optimal annealing temperature = 56°C for all loci.

To test cross‐species amplification of *D. rodriguezii* primers, all 15 SSR loci were amplified in closely related *Daphne* species (Alonso and Herrera, 2011), including leaf material freshly collected from one population of *D. laureola* L. and two to three replicates from herbarium samples for *D. cneorum* L., *D. gnidium* L., *D. mezereum* L., and *D. oleoides* Schreb. (Appendix 1). Rather than testing multiple samples in a closely related species, our aim was to test the cross‐amplification of loci using a broader taxonomic coverage. PCR followed the same conditions previously described for *D. rodriguezii*, but annealing temperatures were chosen based on a temperature gradient protocol ranging from 50–65°C for each primer and *Daphne* species (see Appendix 2). PCR products were visualized on a 1.5% agarose gel stained with GelRed (Biotium Inc., Fremont, California, USA) and sequenced on an ABI PRISM 3130xl Genetic Analyzer sequencer using a GeneScan 500 LIZ Size Standard (Applied Biosystems, Waltham, Massachusetts, USA). GeneMarker 2.4.0 software (SoftGenetics, State College, Pennsylvania, USA) was used for visualizing the electropherograms and performing allele scoring.

The software GENETIX (Belkhir et al., 2001) was used to obtain the number of alleles per locus and estimates of observed and expected heterozygosities. Tests for linkage disequilibrium and potential deviations from Hardy–Weinberg equilibrium following a sequential Bonferroni correction for multiple tests were run on GENEPOP 4.7.0 (Rousset, 2008). MICRO‐CHECKER 2.2.3 (van Oosterhout et al., 2004) was used to assess the presence of null alleles at each locus and population.

At the population level, the number of alleles per locus ranged from one to 11 (Table 2). The level of observed heterozygosity ranged from 0.000 to 0.952, and the level of expected heterozygosity ranged from 0.000 to 0.854 (Table 2). Three loci (Dro025, Dro035, Dro073) were fixed, or nearly so, for a single allele per population. The remaining 12 loci showed substantial levels of polymorphism, with a mean of 8.3 alleles per locus. Only one locus (Dro078) showed significant deviation from Hardy–Weinberg equilibrium after sequential Bonferroni correction in population B, most probably because this was the only combination of locus and population for which null alleles were clearly identified by MICRO‐CHECKER. Significant (*P* < 0.001) linkage disequilibrium was found between loci Dro046 and Dro124, but only for population B.

**Table 2:** Genetic properties of the 15 microsatellite markers tested in two populations of *Daphne rodriguezii*.^a^

Locus	Population A (*n* = 22)				Population B (*n* = 20)			
	*N*	*A*	*H*_o_ ^b^	*H*_e_	*N*	*A*	*H*_o_ ^b^	*H*_e_
Dro012	21	9	0.809	0.851	20	6	0.750	0.772
Dro019	22	5	0.636	0.674	20	3	0.450	0.359
Dro025	20	2	0.100	0.095	20	1	0.000	0.000
Dro028	22	5	0.772	0.700	20	6	0.800	0.766
Dro034	21	8	0.630*	0.810	19	8	0.700	0.791
Dro035	20	1	0.000	0.000	20	1	0.000	0.000
Dro041	22	6	0.727	0.736	20	7	0.750	0.741
Dro042	20	11	0.900	0.778	20	10	0.750	0.821
Dro046	22	7	0.500	0.593	20	3	0.450	0.563
Dro048	22	9	0.600	0.751	20	7	0.850	0.805
Dro073	21	1	0.000	0.000	18	1	0.000	0.000
Dro078	22	11	0.772	0.824	20	10^c^	0.550***	0.826
Dro113	22	6	0.772	0.747	20	5	0.600	0.728
Dro114	21	11	0.801	0.854	19	6	0.400	0.441
Dro124	21	11	0.952	0.844	20	8	0.600	0.646

Note

*A* = number of alleles detected across *D. rodriguezii* samples; *H*_e_ = expected heterozygosity; *H*_o_ = observed heterozygosity; *n* = number of samples tested; *N* = number of samples with successful amplifications.

a Locality and voucher information are provided in Appendix 1.

b Asterisks indicate significant deviation from Hardy–Weinberg equilibrium after Bonferroni correction (*P < 0.05, ***P ≤ 0.001).

c Presence of null alleles.

In addition, this panel of microsatellites rendered positive amplifications in a minimum of seven loci per species (Table 3). The limited availability of herbarium samples per species precluded a clear assessment of the levels of polymorphism detected with these markers, but for some species (*D. laureola*,* D. cneorum*,* D. oleoides*), even relatively low sample sizes revealed that at least half of the amplified loci exhibited more than one allele (Table 3).

**Table 3:** Cross‐amplification of microsatellite markers developed for *Daphne rodriguezii* in five closely related species.^a^

Locus	*D. laureola* (*N* = 5)	*D. cneorum* (*N* = 2)	*D. gnidium* (*N* = 2)	*D. mezerum* (*N* = 1)	*D. oleoides* (*N* = 2)
Dro012	—	279	255	251	257, 271
Dro019	—	—	247	—	—
Dro025	156	136, 151, 163	152, 156, 168	156	154, 156, 162, 164
Dro028	190	235, 239, 253, 289	198	—	201, 207, 213
Dro034	—	—	—	—	—
Dro035	204, 206	205, 207	192	204, 206	204, 206
Dro041	181, 184	184	184	—	184
Dro042	314, 317, 320	—	164	290	180
Dro046	266	—	173, 179	—	—
Dro048	—	—	—	—	—
Dro073	205, 217	205	205, 217	205, 217	205
Dro078	—	148, 157, 173	—	—	—
Dro113	—	—	—	122	107
Dro114	—	—	—	212	—
Dro124	155	—	—	—	233, 253

Note

— = unsuccessful amplification; *N* = number of samples tested for each species.

a Voucher and locality information are provided in Appendix 1.

## CONCLUSIONS

The set of microsatellites characterized for *D. rodriguezii* is a powerful, cost‐effective tool for detecting substantial levels of genetic variation using a relatively low number of multiplexed reactions, even in small populations. Such a genetic resolution will allow us to assess parentage relationships in forthcoming studies on fine‐scale genetic structure. Additionally, the successful rates of cross‐amplification of these loci suggest that population genetic studies with these markers could be easily extended to other closely related *Daphne* species.

## AUTHOR CONTRIBUTIONS

C.G.‐V. and A.T. planned the study and collected plant tissue, J.C.I. and C.G.‐V. conducted laboratory work and allele scoring, and C.G.‐V. performed the analyses and wrote the manuscript, with input from J.C.I. and A.T.

## Data Availability

The primers and microsatellite sequences developed in this study have been deposited in GenBank (accession numbers MK507747–MK507761; Table 1). Raw sequence library data were deposited in the Short Read Archive of the National Center for Biotechnology Information (NCBI) (BioProject accession number: PRJNA523502).

## APPENDIX 1 Voucher and location information for species and populations used in the characterization of microsatellite markers for *Daphne rodriguezii* and related species.

**Table:**

Taxon (Population)	Voucher specimen accession no.^a^	Collection locality	Geographic coordinates	*N*
*Daphne rodriguezii* Texidor (popA)	JBAG8300	Colom, Menorca	39°57.5′N, 04°16.9′E	22
*Daphne rodriguezii* (popB)	JBAG8301	Mesquida, Menorca	39°54.5′N, 04°17.0′E	20
*Daphne cneorum* L.	JBAG656	Valle del Soba, Cantabria	43°09.5′N, 03°34.1′W	3
*Daphne gnidium* L.	JBAG877	Dumbría, La Coruña	43°00.9′N, 09°07.4′W	3
*Daphne laureola* L.	JBAG8299	Ponga, Asturias	43°12.7′N, 05°05.5′W	5
*Daphne mezereum* L.	JACA78470	Canfranc, Huesca	42°42.2′N, 00°34.1′W	2
*Daphne oleoides* Schreb.	JBAG384	La Rapa, Granada	37°20.1′N, 02°50.2′W	2

Note

*N* = number of individuals initially assayed (some herbarium samples did not provide clear amplifications and were not used for polymorphism testing; see Table 3).

a All herbarium specimens are deposited at the Jardín Botánico Atlántico herbarium (JBAG), Asturias, Spain, including one donation from the Instituto Pirenaico de Ecología herbarium (JACA), Jaca, Spain.

## APPENDIX 2 Optimal PCR annealing temperatures (◦C) used for cross‐species amplification of microsatellite markers developed for *Daphne rodriguezii* in five closely related species.

**Table:**

Locus	*D. cneorum*	*D. gnidium*	*D. laureola*	*D. mezereum*	*D. oleoides*
Dro012	55.7	52.6	MB	52.6	60.2
Dro019	—	55.7	—	—	—
Dro025	59.0	59.0	59.0	59.0	59.0
Dro028	50.0	60.0	60.0	—	50.0
Dro034	—	—	MB	—	—
Dro035	63.6	59.0	59.0	59.0	59.0
Dro041	51.0	50.0	50.0	—	50.1
Dro042	—	55.7	60.0	51.2	51.2
Dro046	—	51.2	50.1	—	—
Dro048	—	—	—	—	—
Dro073	59.0	59.0	59.0	59.0	59.0
Dro078	50.1	—	MB	—	—
Dro114	MB	MB	MB	60.0	MB
Dro113	—	—	—	59.1	60.2
Dro124	—	—	52.6	—	55.7

Note

— = unsuccessful amplification; MB = multiple bands.
